# Artificial skin through super-sensing method and electrical impedance data from conductive fabric with aid of deep learning

**DOI:** 10.1038/s41598-019-45484-6

**Published:** 2019-06-20

**Authors:** Xi Duan, Sebastien Taurand, Manuchehr Soleimani

**Affiliations:** 1 Engineering Tomography Laboratory, Department of Electronic and Electrical Engineering, University of Bath, Claverton Down, BA2 7AY UK; 2 SICOM, PHELMA, Grenoble INP, 3 Parvis Louis Néel, 38000 Grenoble, FR France

**Keywords:** Mechanical engineering, Soft materials

## Abstract

Sense of touch is a major part of man’s communication with their environment. Artificial skins can help robots to have the same sense of touch, especially for their social interactions. This paper presents a pressure mapping sensing using piezo-resistive fabric to represent aspects of the sense of touch. In past few years’ electrical impedance tomography (EIT) is considered to be able offer a good alternative for artificial skin in particular for its ease of adaptation for large area skin compared to individual matrix based sensors. The EIT has also very good temporal performance in data collection allowing for monitoring of fast responses to touch stimulation, enabling a truly real time touch sensing. Electromechanical responses of a conductive fabric can be exploited using EIT to create a low cost and large area touch sensing. Such electromechanical properties are often very complex, so to improve the imaging resolution and touch visibility an artificial intelligent (AI) was used in addition to the state of the art spatio-temporal imaging algorithm. This work demonstrates a step towards an integrated seamless skin with large area sensing in dynamical settings, closer to natural human skin’s behaviour. For the first time a dynamical touch sensing are studies by means of a spatio-temporal based electrical impedance tomography (EIT) imaging on a conductive fabric. The experimental results demonstrated the successful results by a combined AI with dynamical EIT imaging results in single and multiple points of touch.

## Introduction

Over the last two decades, there are various types of artificial skin based on different touch sensing techniques have been developed^1^. Piezoresistive sensor measures the resistance changes of the elastomer, foam, conductive carbon ink or other conductive material while the pressure force applied directly^2,3^. This well-structured touch sensor is commonly used on robotic hands, gives high resolution and low cost^4,5^. However, the high power consumption, low repeatability, and large hysteresis are some of the drawbacks of piezoresistive sensors. At the same hand, capacitive sensors have been widely used in robotic since they have relatively high sensitivity and can give good spatial resolution, but the structure of the sensors is more complicated due to its sandwich design and the capacitors arrays, which easily causes noise and interference^6^. Optoelectronic sensors use optical technology; the pressure can be detected by changing in light intensity or spectrum. It has the advantages of large sensing range, high spatial resolution, immune to electromagnetic interference and fast response, but they are huge in size, required high power supply^7,8^. Other tactile sensors using magnetic, piezoelectric, and ultrasonic are introduced and summarised in^4,9^.

Electrical impedance tomography (EIT) as an imaging technique, can determine the interior conductivity distribution of the object by only using measurements from electrodes placed on its boundary. The utilizations of the EIT have been successfully in biomedical, geophysical and industrial fields, it has been used for such as the brain and thorax monitor, liquid mixing and flow analysis because of the non-destructive property. Instead of the human body or other target, if a conductive material that will respond to local conductivity of permittivity changes is used, a pressure mapping imaging system or a touch sensing sensor can be created. Commonly, a large-scaled sensor array for robotic skin consists numbers of interior wires or electrodes, which can easily cause electromagnetic noise and affect on the flexibility and stretchability of the skin. Where, the EIT based fabric sensor were introduced to overcome this issue. The first tactile sensor based on EIT without wire and sensing elements in its region was presented in^10^, the rubber response to pressures with local changes in conductivity. To realise the light-weight, low-cost and stretchable artificial skin, single-layered conductive fabrics were used in^11^ and^12^. A highly stretchable tactile distribution sensor was used for smooth surfaced humanoids^13^. A tactile distribution sensor which enables stable measurement under high and dynamic stretch was also^14^. They demonstrated an improved EIT-based fabric sensor which can map local pressure. However, the image resolution of fabric EIT is experiencing low quality problem. Important information is surrounded by noise and other impact.

In this paper, the EIT is used for mapping touch over time and space. The work presents underlying mathematical framework of EIT-based fabric sensor, analyses some major sources of error and perform various dynamic experiments and improve image quality using deep learning for the first time.

## Measurement System

**Figure 1:**
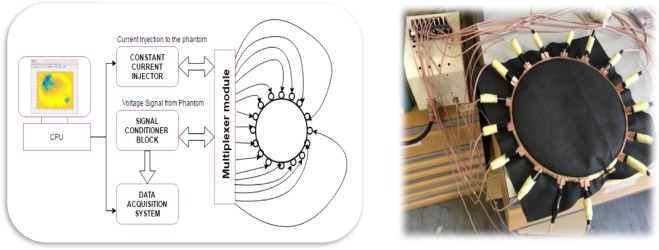
A general EIT system.

Electrical impedance tomography (EIT) aims to reconstruct image of a conductivity distribution inside the testing object. EIT systems measure boundary voltages according to constant, low frequency and multiple injection currents to reconstruct cross-sectional images of the conductivity distribution^15^. A typical EIT system is shown in Fig. 1. All the proposed EIT systems are based on the basic methodology which are: (1) the data collection of a set of independent transfer electrical impedances. (2) The stable solutions of forward and inverse problems in order to reconstruct images with high quality^16^. To reconstruct EIT images in general geometrical settings, all functions are required which are described in details: a system for measuring the surface geometry, a mesh generator to produce a mesh based on the surface information, code for computing the finite element approximation and an algorithm to solve the inverse problem. Also, tools to display the results and also to analyse the images are required^17^. Therefore, 4 key parts of the EIT system are shown below, The basic EIT system has four main components: (i) EIT-Instrumentation, (ii) Electrode Array or EIT Sensors, (iii) PC with Reconstruction Algorithm, and (iv) Subject under test (SUT).

## Method

**Figure 2:**
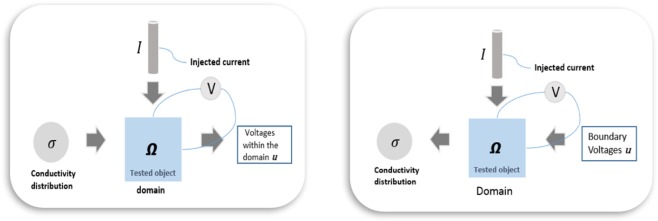
EIT forward (left) and inverse (right) problems.

To generate EIT images one needs to solve the forward model, which is physical model of measurement processes and the inverse problems of identifying the conductivity maps by the boundary measurements. Figure 2 shows these two mathematical problems in diagrammatic format.

The forward problem of EIT is determining the voltage distribution of a known domain by a given current and a specified conductivity distribution. The finite element method (FEM) is a numerical technique for solving practical differential equations, finite element meshing is utilized to discrete the domain and to calculate the voltage as a forward solver^18^. For an object occupy a two-dimensional region Ω with its boundary ∂*Ω*, electrodes *e*_1_, l = 1, 2, 3, …, L on the boundary. A current I with an angular frequency ω is applied to an adjacent electrodes pair, by calculating the divergence of both sides of the Maxwell equations, the following equation is obtained:

1 $$\nabla \cdot (\sigma \nabla u)=0$$

with the boundary condition

2 $$\sigma (\partial u/\partial n)=J$$

The boundary condition was introduced because of electrodes are inevitably mounted on the boundary of the sensing field^19^. Where *σ* and *u* are the distributions of conductivity and potential. And *J* is the electric current density on the boundary, ∂/∂*n* is the normal derivative to the boundary.

To get the current j, appropriate boundary condition need to be considered to build an accurate model for EIT. In this study we use the complete electrode model (CEM), which considers both the shunting effect of the electrodes and the contact impedance^20^. Given the CEM equation:

3 $$u+{z}_{l}\cdot \sigma \frac{\partial u}{\partial n}={V}_{l},\,x\in {e}_{l},\,l=1,2,\ldots ,\,L,$$

4 $${\int }_{{e}_{l}}\sigma \frac{\partial u}{\partial n}dS={I}_{l},\,x\in {e}_{l},\,l=1,2,\ldots ,L,$$

5 $$\sigma \frac{\partial u}{\partial n}=0,\,x\in \partial {\rm{\Omega }}/{u}_{l}^{L}{e}_{l}$$

where *z*_*l*_ indicates the contact impedance between the *l*th electrode and the tissue, *n* is the outward normal, *V* is the voltage and *I* is the current. Equation (5) indicates behaviour of the non-electrode boundary. In order to ensure the existence and the uniqueness of the solution, the law of the conservation of the charge is applied:

6 $$\sum _{l=1}^{L}\,{I}_{l}=0$$

The forward operator *F* is then defined as a map from the conductivity distribution *σ* to the measured boundary voltage *u*, that is, $\,F(\sigma )=u$.

The inverse problem in EIT can be defined as the recovery of a change in conductivity Δ*σ* from a change in measured boundary voltage Δu. To solve this inverse problem, the matrix described the mapping between Δu on the boundary and Δ*σ* in the interior is required. And we call this matrix as Jacobian Matrix, where can be written as ${\rm{\Delta }}u={\rm{A}}\,{\rm{\Delta }}{\rm{\sigma }}$. Based on the Reciprocity theorem, A is computed by the Fréchet derivative of u with respect to *σ*^21^. The regularization method used in this study is spatiotemporal total variation (ST-TV)^22^, as in dynamic fabric EIT, consecutively images are expected to show a strong correlation, where spatial and temporal gradient domains are involved based on total variation^23^. states that spatiotemporal total variation problem as:

7 $$arg\mathop{\min }\limits_{{\rm{\Delta }}\sigma }{\Vert {\nabla }_{x,y}{\rm{\Delta }}\sigma \Vert }_{1}+{\Vert {\nabla }_{t}{\rm{\Delta }}\sigma \Vert }_{1}s.t.{\Vert \tilde{A}{\rm{\Delta }}\sigma -{\rm{\Delta }}u\Vert }_{2}^{2}\le \delta $$

where two terms of the function represent spatial and temporal behaviour, and where Δ*σ* represents a 3D (*x*, *y*, *t*) conductivity distribution and $\tilde{A}$ is an augmented Jacobian operating on a acts in a frame-by-frame basis^24^.

## Results and Discussion

This work focuses on a Pressure Mapping Imaging based on EIT. The special sensor used is built up by a conductive fabric and a wooden circle frame with 16 electrodes on boundary. The conductive stretchable fabric LTT-SLPA used in the experiment was manufactured by Eeonyx Corp with the ability to stretch in both direction, surface resistivity (conductivity) changes as it is stretched or compressed, made by stretchy nylon/spandex and coated with a long-lasting conductive formulation, which gives a surface resistivity in the range of 10^4^ to 10^7^ ohm/sq. Two of the electrodes are used to input a constant current into the conductive fabric, other electrodes will get an initial voltage reading. And then, pressuring the conductive fabric over the circular sensor area, electrodes on boundary will generate a new voltage reading. Using this new reading minus initial voltage reading to get voltage difference (Δ*V*). The voltage difference is used to image pressure map by MATLAB processing. We conducted several experiments and two sets of experiments are shown here for comparative study.

### One-object test

**Figure 3:**
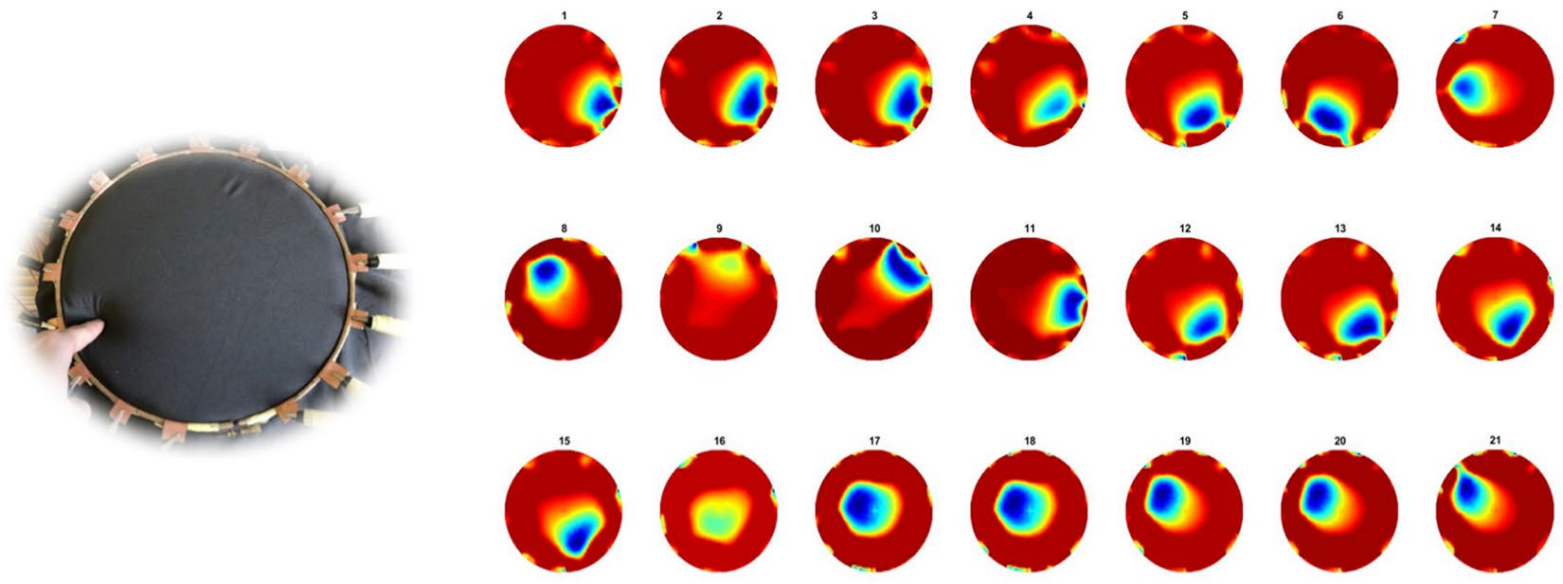
A single pressure point moving counter clockwise and then diagonally towards the center.

In this test, one finger was used to apply the pressure on fabric over the circular fabric sensor area (as shown in Fig. 3(a)). The background data was collected first, and then apply force from first test point. The pressure point was moving anticlockwise near boundary and moving towards center after one turn. KHU Mark 2.5 data collection system (Kyung Hee University, South Korea) is used with a 50 frames per second temporal. But when it comes to electromechanical sensing which involves mechanical reposes of the fabric this 50 frames per second cannot be achieved. The temporal resolution becomes around 1 frame/sec. In this case 397 sets of scan data were collected.

Figure 3(b) shows 21 snap of reconstructed images for a single finger touch going anticlockwise and from frame 13 moving towards top left and then through the center.

### Two-object test

**Figure 4:**
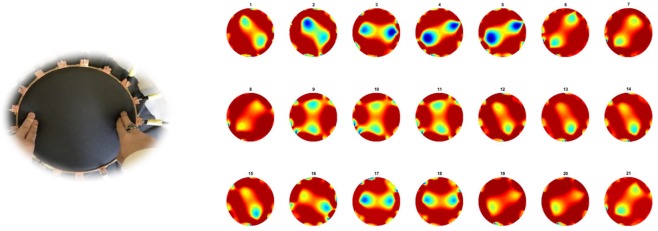
Double pressure points rotating clockwise.

In this experiment, the same approach as test one, two pressure points applied at the same time and moving clockwise. In this case 252 sets of data were collected. Figure 4 shows clockwise movement of two finger touch points.

Temporal image reconstruction using state of the art TV algorithm performed very well, taking advantage of time correlation between frames of data. This includes ease of detection of pressure points in central area of fabric, which is always seen an issue for large area fabric EIT testing. There are still some artefacts remaining in those images and if we revert to a single frame by frame reconstruction (not taking advantage of temporal correlations), then these noises are more severe. Next section aims to address these issues with help from arterial intelligent.

### Image processing – Deep learning

Since the beginning of EIT research, it is known that the shape mismatch and electrode position uncertainty are key sources of errors in imaging reconstruction^22^. In the most industrial applications and phantom simulations, the electrodes are stably mounted around boundary, reconstruction for these cases will not affected by the movement of electrode. However, in fabric EIT, the natural of fabric causes the shape mismatch when pressure applied.

**Figure 5:**
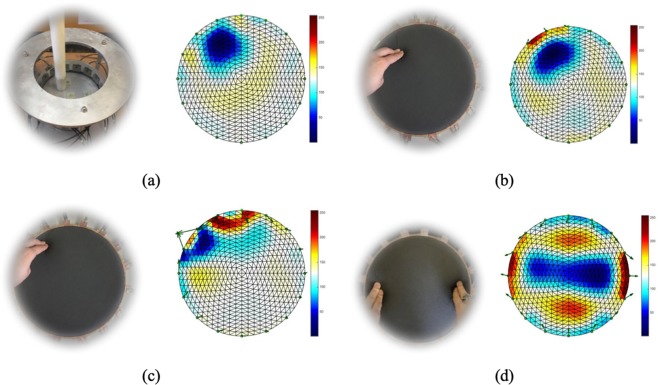
Top: reconstructed images for (**a**) phantom data with one non-conductive inclusion and (**b**) fabric data with one pressure point in top left. Bottom: reconstructed images for fabric data with (**c**) one pressure point close to boundary and (**d**) two pressure points. A pressure point in central area is challenging due to lower EIT sensitivity but has potentially less effect on electrode movement.

The arrows in Fig. 5 indicated each electrode’s movement. In the image of phantom data, there is no electrode movement which gives a fairy good reconstruction result. For the fabric data, reconstructed images are damaged by the noise caused by electrode movement. The closer of the pressure point is to the boundary, the more the electrode will be affected around pressure point, the greater amplitude of the movement by these electrodes gives more collapse in image reconstruction. Also, the area around pressure point are blurred due to the net structure of the fabric, especially when there are more than one pressure points, the separation can be difficult like shown in Fig. 5(d).

**Figure 6:**
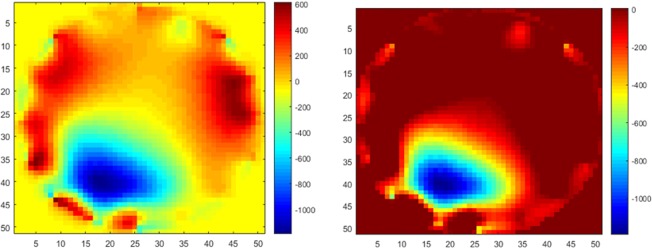
Passage from a raw EIT image (left) to a thresholded image (right).

There is no way to change the elastic structure of the fabric, and this is a great advantage in applications such as artificial skin and wearable monitoring. Therefore, implies post image processing is necessary to improve image quality and visibility caused by these inevitable physical phenomena. Figure 6 shows a reconstructed image and a typical thersholding, the initial image and include noises, but a systematic error such as the ones shown in Fig. 5 due to electrode movement could be removed by AI training.

The aim is to remove the electrodes artefacts which can be done with deep convolutional neural network. In general, a neural network consists of an input layer, a set of hidden layers formed of neurons and an output layer. A neuron contains all input information *x*, where *x* is a vector, interact with weights *w* (learnable parameter). The result of weighted sum *xw* passes through an activation function *f*. The output of the neuron is thus $y=f\,(\sum {x}_{i}{w}_{i})$.

**Figure 7:**
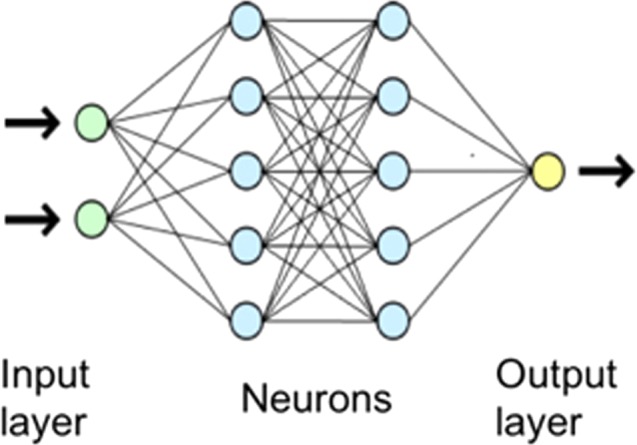
A regular 3-layer neural network.

In the case of a classical neural network, each neuron takes all values in previous layer as input like in Fig. 7. This means for a network with an image as input and made up of approximately 10 to 20 layers, there will be millions of weights to learn at the same time and the number of calculations is huge. In fabric EIT images, the size is modest and yet the number of calculations remains very important. Which convolutional neural network is rather good when treating images due to the lower calculation complexity by parameter sharing, neurons do not take into account of all pixels as input. And this type of calculations considers neighborhood in images^25^.

**Figure 8:**
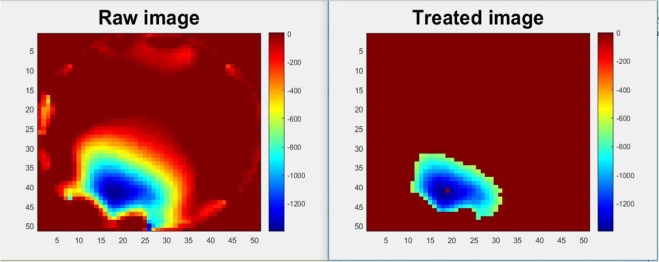
A set of raw and treated image.

A learning base is created by an input of a set of noisy images and an output of all cleared images. The network is updated with the information through learning, which is modifying weights *w* until reaching the desired result, and then, a set of unreleased images can be sent to the network to improve visibility. The learning base has to be as robust as possible. In our case, the input is images representing pressure point that travel all accessible areas of the fabric with a significant noise related to the electrodes. The output is the improved image that we want, in general, the perfect image is not known, that is why in this learning the “perfect” images have been created. An Example of producing a “perfect” image is shown in Fig. 8. A boundary suppression applied to remove electrode’s noise, and large size of blobs were kept only values greater than 55% of the maximum amplitude since even a small pressure point can produce a large blob due to the deformation of the material. In additional, small blobs that less than 15 pixels of area was removed.

**Figure 9:**
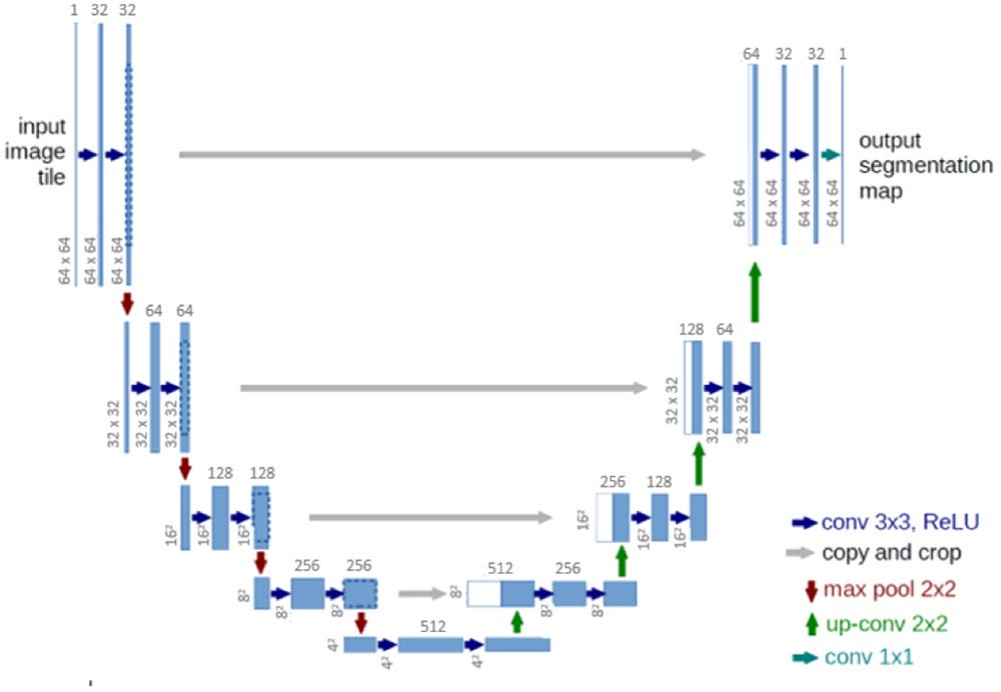
Structure of a convolutional neural network of U-net architecture.

In this image training, a U-net convolutional neural network implemented with Tensorflow (https://www.tensorflow.org/) is used, the structure of this network is shown in Fig. 9. This type of network is commonly used in biomedical image segmentation, also has good results during classification problems and image reconstruction^26^.

#### Training 1

The one object dataset training from experiment one is composed of 439 pairs of images where one pair of image contains a raw image and the image wanted. Once the training is done, new images with noise artefacts can be sent into the neural network, the output being an image hopefully improved. The proposed network could be simplified to a smaller network as the overall task in this problem of 2D and time is less demanding than some other challenging problems U-net can deal with.

Measurement of image clearing quality is done by two approaches.

- With the formula of mean squared error:
  $$MSE=E[{(image-imag{e}_{wanted})}^{2}]$$
- And simply by exanimating the final image keeping in mind the dynamic of real experience.

**Figure 10:**
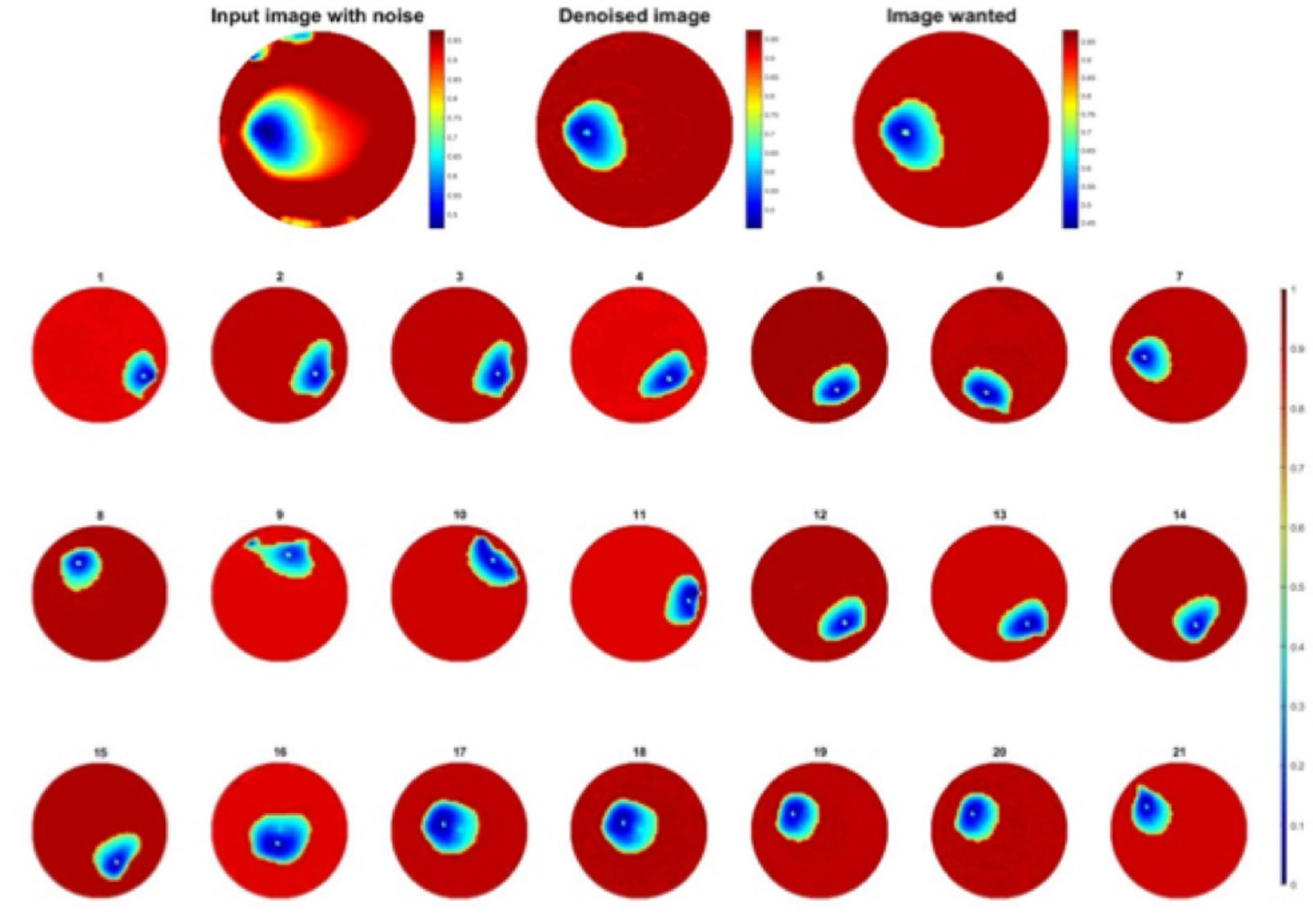
(**a**) One frame of single pressure point test and (**b**) 21 corresponding frames improved through the network trained with one object dataset.

The test of the network was done with the initial training data since almost each possible position is taken in this set, the set is exhaustive. Training results are shown in Fig. 10, electrodes noise is almost suppressed, 21 corresponding frames in Fig. 3b are greatly improved. The pressure points are clearly detected, and they are almost near “perfect” images since the test dataset is the same as training set.

Second test to check the robustness of the learning is to see how two objects images are improved through one object trained network. Test was made on 273 images from experiment two.

**Figure 11:**
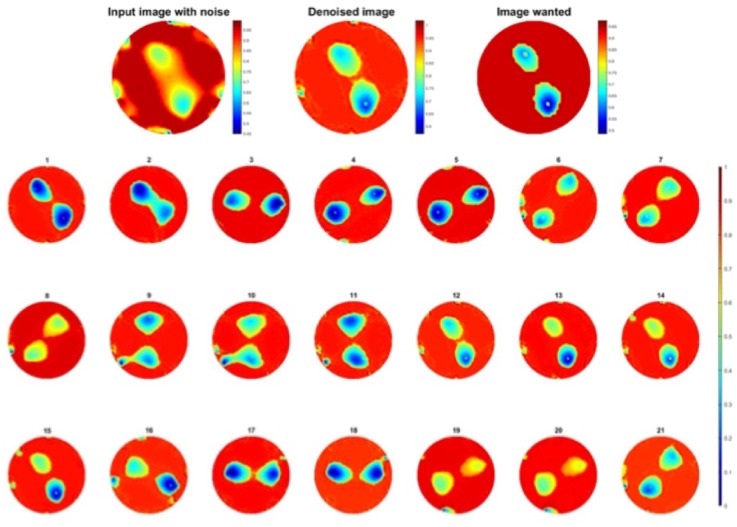
(**a**) A example and (**b**) 21 corresponding improved images obtained for two objects passed through a one object trained network.

**Figure 12:**
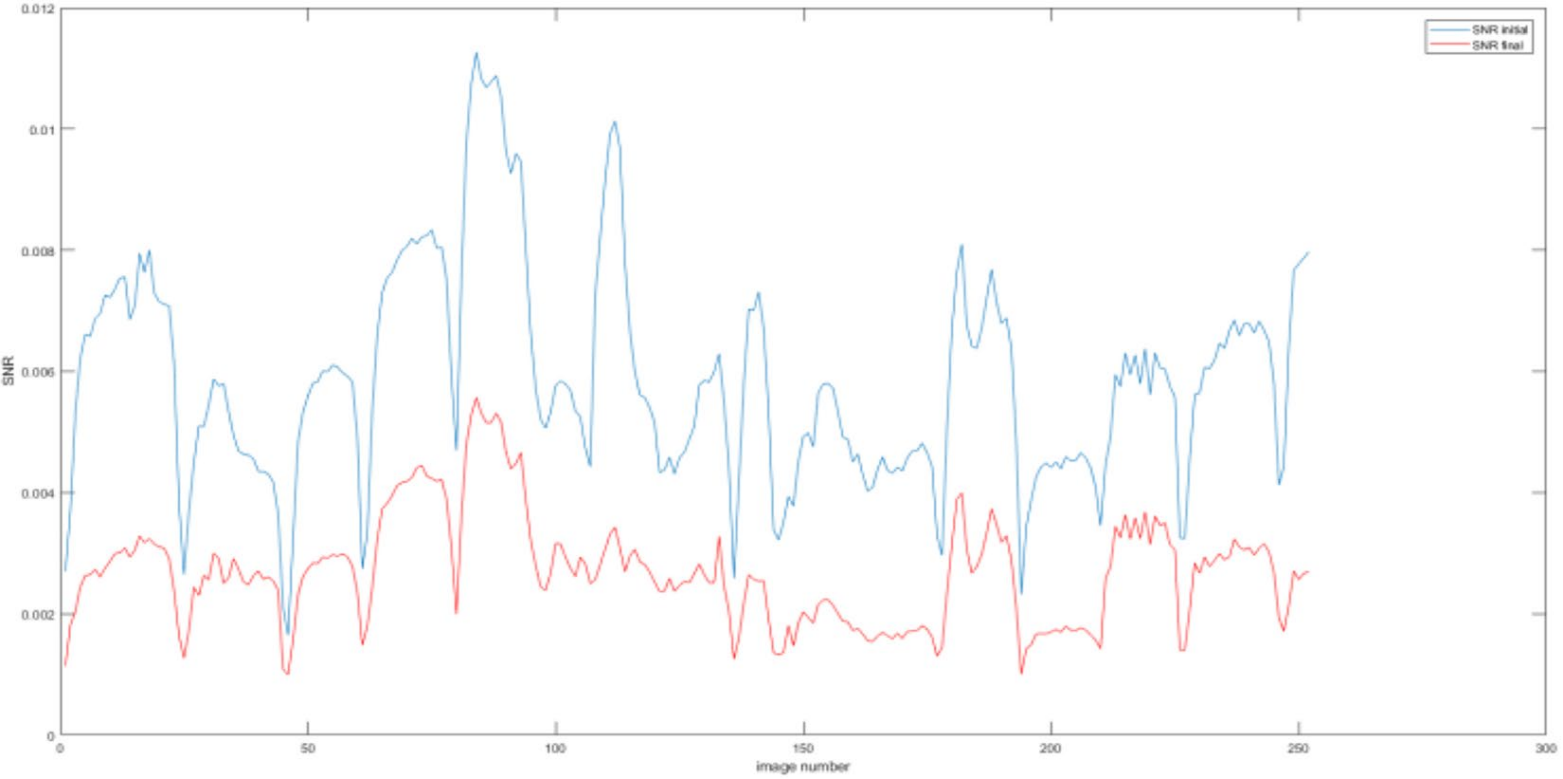
Comparison of MSEs at the input (in blue) and the output (in red) of the network trained with one object images and tested with two objects images.

Results are shown in Fig. 11, compare to the images before processing in Fig. 4, pressure points are clearly detected, most noise caused by electrodes and fabric deformation are eliminated. Also to be noted that separation between objects is very well observed leading to better resolution. MSEs are calculated in Fig. 12, the error of the output has been reduced by more than half.

#### Training 2

The other approach is to train the network with two objects images. In this experiment the dataset of training was using the test set in last section, composed of 273 pairs of images, from two-object experiment. Once the training done the first test was to look at the improvement of image quality for two objects images.

**Figure 13:**
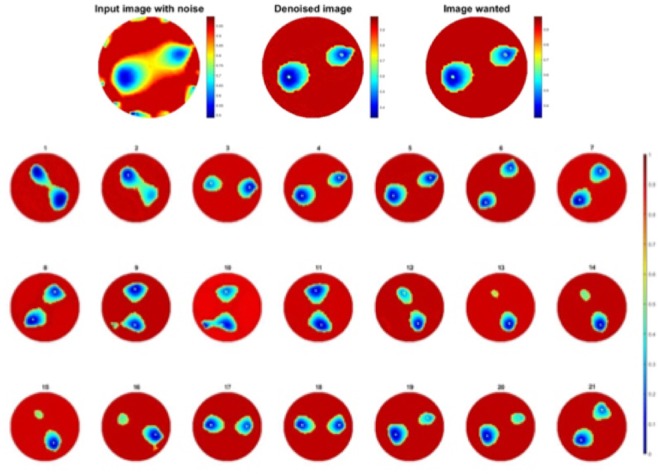
Improvement of two objects image passed through two objects trained network.

Results are again really good, shown in Fig. 13, electrodes noise is mostly removed and separation between objects is well done.

Since the test dataset was the same as training dataset in last experiment, the second experiment is using one object dataset of 439 pairs of images which is the same one as for the one object learning in *Training 1*.

**Figure 14:**
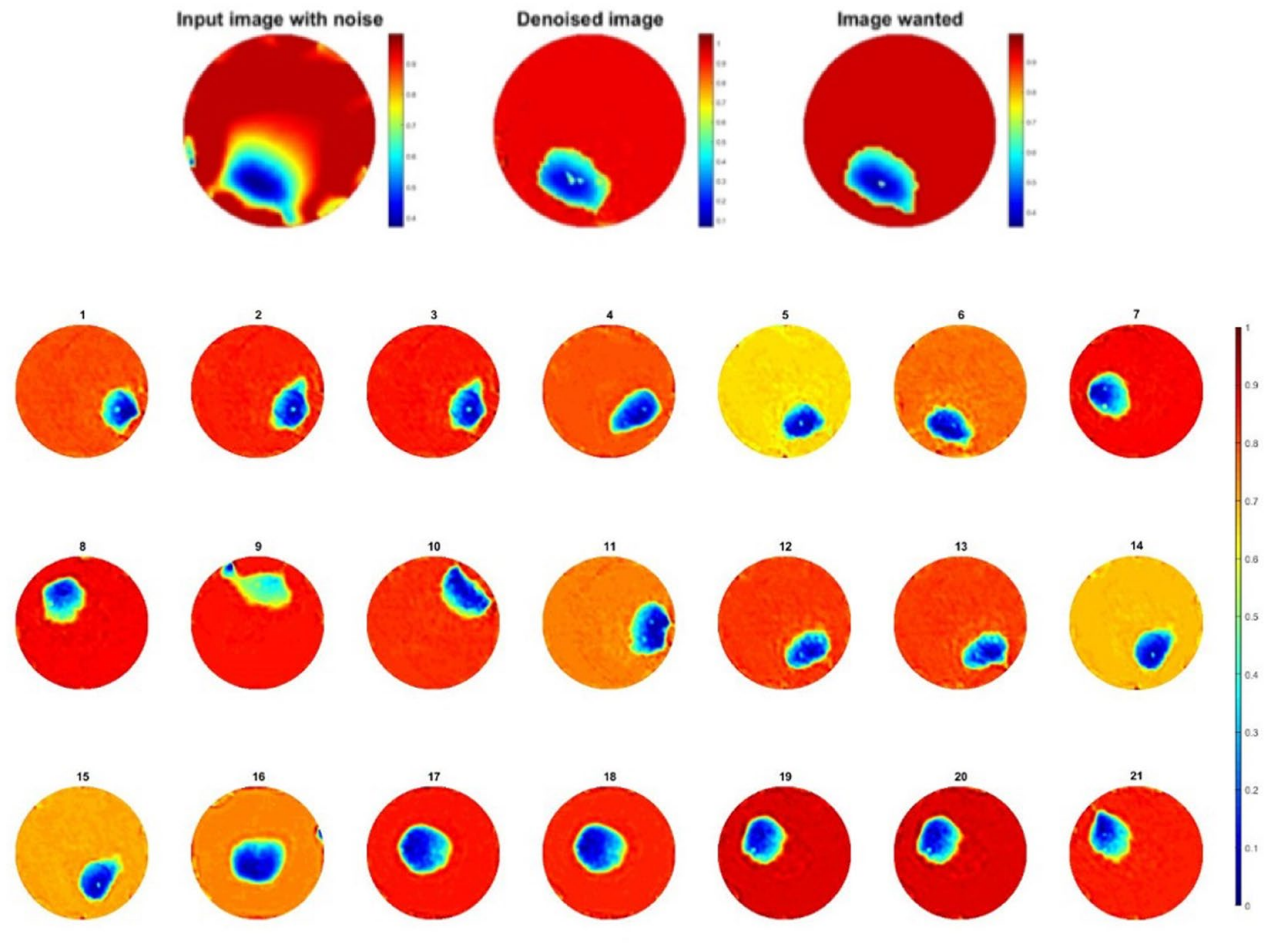
Result of one object image improvement passing through two objects trained network.

**Figure 15:**
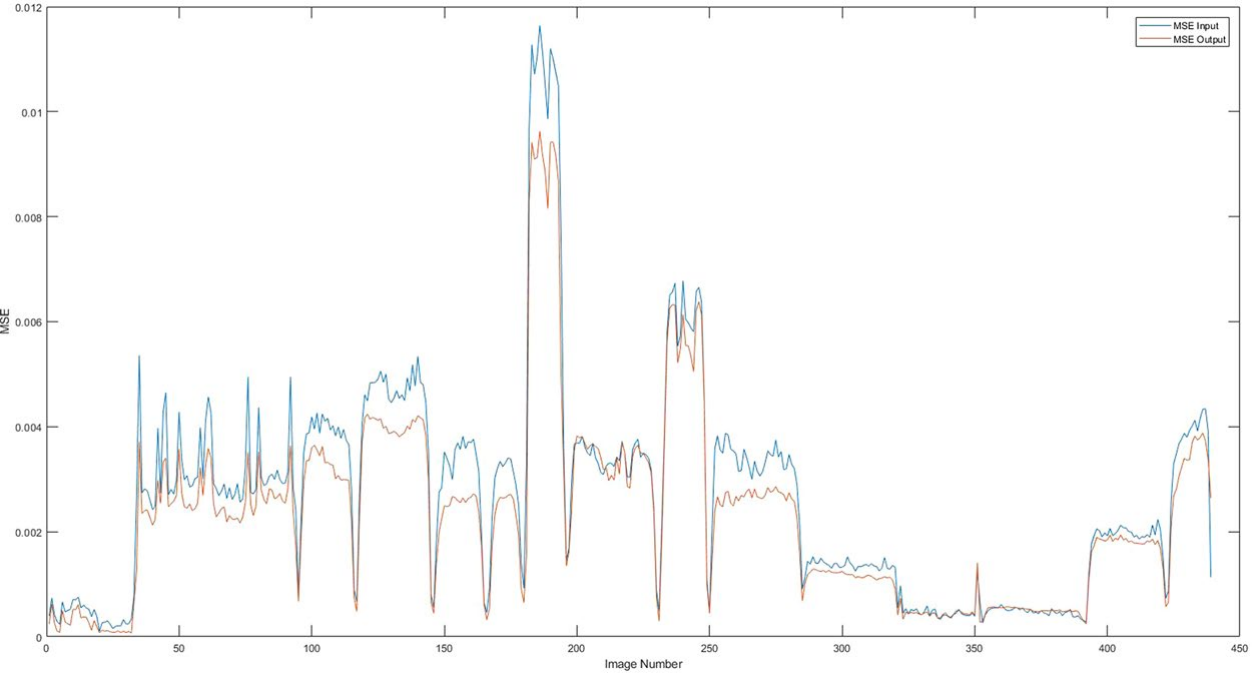
Comparison of MSEs at the input (in blue) and the output (in red) of the network.

This is an interesting observation where the neural net was used to train by using two pressure points data and tested against a single point experiment, the results were very good shown in Fig. 14. The MSE of the output is reduced as desired shown in Fig. 15. Compare to the images in Fig. 3(b), there is a huge improvement in visibility, the amplitude and location of the pressure point is more specific, almost all error caused by electrodes movement are eliminated.

A conductive fabric behaviour is complex in both resistive and reactive terms under and applied AC current, because of the capacitances produced by the interactions of the conductive yarns and air gaps. A stretchable conductive fabric shows an even more complex behaviour. Due to complex nature of this sensing the deep learning approach shown in this paper actually does more than an image post processing, it overcome some of these model uncertainties that are extremely hard to physical model.

## Conclusion

An ideal material used in EIT system for artificial skin would be light-weight, have continuous and homogeneous conductivity, low-cost, local conductivity changes in response to touch or pressure and will not be affected by stretching. The fabric used in this study is conductive, the conductivity changes as it is stretched, and it is light-weight and low-cast as wanted. This simplicity of sensor comes with some complex electromechanical behaviour which is not easy to model in forward model. Hence the use of the deep learning is justified in this application. In previous study, pressure or touch could be detected by fabric EIT system statically. The dynamic behaviour of the fabric EIT system were presented in this paper for the first time. The experimental shows good results of temporal performance in data collecting and generated a movie like pressure mapping at the same time. There are still some major errors caused by electrode movement and other inevitable shape-shift of the fabric in reconstruction images using spatiotemporal total variation algorithm. The hysteresis behaviour was shown during experiment, motivating future studies in time-related response of the fabric EIT system. In training stage, the results of test data set are going through trained net-work (using independent data set) are impressive in error reduction for all cases shown here. The way to improve image visibility and reduce these errors through neural network is effective and has the advantage of being autonomous.
